# Discrepancy between WISC-III and WISC-IV Cognitive Profile in Autism Spectrum: What Does It Reveal about Autistic Cognition?

**DOI:** 10.1371/journal.pone.0144645

**Published:** 2015-12-16

**Authors:** Anne-Marie Nader, Patricia Jelenic, Isabelle Soulières

**Affiliations:** 1 Psychology Department, University of Quebec in Montreal, Montréal (QC), Canada; 2 Research center of Institut universitaire en santé mentale de Montréal, Rivière-des-Prairies Hospital, Montreal (QC), Canada; Ecole Normale Supérieure, FRANCE

## Abstract

The cognitive profile and measured intellectual level vary according to assessment tools in children on the autism spectrum, much more so than in typically developing children. The recent inclusion of intellectual functioning in the diagnostic process for autism spectrum disorders leads to the crucial question on how to assess intelligence in autism, especially as some tests and subtests seem more sensitive to certain neurodevelopmental conditions. Our first aim was to examine the cognitive profile on the current version of the most widely used test, the Wechsler Intelligence Scales for Children (WISC-IV), for a homogenous subgroup of children on the autism spectrum, i.e. corresponding to DSM-IV diagnosis of “autism”. The second aim was to compare cognitive profiles obtained on the third edition versus 4^th^ edition of WISC, in order to verify whether the WISC-IV yields a more distinctive cognitive profile in autistic children. The third aim was to examine the impact of the WISC-IV on the cognitive profile of another subgroup, children with Asperger’s Syndrome. 51 autistic, 15 Asperger and 42 typically developing children completed the WISC-IV and were individually matched to children who completed the WISC-III. Divergent WISC-IV profiles were observed despite no significant intelligence quotient difference between groups. Autistic children scored significantly higher on the Perceptual Reasoning Index than on the Verbal Comprehension Index, a discrepancy that nearly tripled in comparison to WISC-III results. Asperger children scored higher on the VCI than on other indexes, with the lowest score found on the Processing Speed Index. WISC-IV cognitive profiles were consistent with, but more pronounced than WISC-III profiles. Cognitive profiles are a valuable diagnostic tool for differential diagnosis, keeping in mind that children on the autism spectrum might be more sensitive to the choice of subtests used to assess intelligence.

## Introduction

Autism is a neurodevelopmental condition characterized by significant heterogeneity of behavioral characteristics, cognitive skills and developmental trajectories[1, 2]. To deal with this heterogeneity, the DSM-5 emphasizes the importance of defining the individual developmental profile of persons on the autism spectrum, including intellectual functioning, as they can present important diagnostic assets and differential tools for intervention. “These specifiers provide clinicians with an opportunity to individualize the diagnosis and communicate a richer clinical description of the affected individuals”[3]. Thus, results on different subtests and index scores derived from intellectual measures are useful information when characterizing strengths and weaknesses among autistic individuals[4].

Although intelligence has long been conceived by the general population as being a stable trait[5, 6], the portrait is less clear in autism due to some indications of variability over time and across assessment instruments. While some previous studies have reported that intellectual functioning in autism remains stable over time[7–11], other authors have shown an improvement of cognition in terms of the overall intelligence quotient (GIQ)[12, 13]. In addition, children with better cognitive abilities have a more stable intellectual trajectory than children with lower intellectual functioning[9, 13]. However, the results of these studies are difficult to compare due to the variety of assessment tools that have been used[10].Furthermore, numerous studies showed a discrepancy between different intelligence tests in the autistic population, a difference not found in the general population. As an example, autistic individuals tend to perform better on the Raven Progressive Matrices, a well established tool assessing complex reasoning, than on the Wechsler scales[14].Moreover, better results have been found on Leiter International Performance Scale than on the Stanford Binet Intelligence Scales-5^th^ edition [15].

Nevertheless, the Wechsler scales remain the most frequently used when assessing intellectual functioning and cognitive abilities in research and clinical settings[6, 16]. A distinctive pattern of performance in individuals with autism has repeatedly been found on the Wechsler Intelligence Scale for Children, 3^rd^ edition (WISC-III) [17]. Indeed, several studies have described a significantly higher Performance IQ (PIQ) than Verbal IQ (VIQ)[18–23], occurring more frequently among the autism spectrum than in normative samples[24, 25]. Different patterns in subtest performance were also encountered among autistic individuals. When compared to other subtest scores, autistic individuals’ performance peaked on the *Block Design* subtest [18, 22, 26–32], and was at its lowest on the *Comprehension* subtest [28, 29, 31, 33–35].Furthermore, their performance pattern is occasionally accompanied by low scores on the *Digit Span* and *Coding* subtests[27, 36].

The fourth edition of the WISC (WISC-IV) [37] introduced significant changes in the subtests and structure of indexes. First, Performance and Verbal IQ scores were replaced with four index scores according to the results of previous factor analyses[38]. Three of the four WISC-III Verbal IQ subtests were retained on the WISC-IV with some item changes. On the other hand, the WISC-IV Perceptual Reasoning Index changed substantially since the last edition and now comprises three subtests: *Block Design* from the previous edition and two new untimed, motor-free, visual reasoning tests (*Picture Concepts* and *Matrix Reasoning*). Therefore, the PRI now has only one timed visual-motor test in contrast to three on the WISC-III. Changes brought to the PRI decrease demands on perceptual-motor abilities, leading to a purer measure of fluid reasoning [16].Studies with children presenting other neurological conditions (i.e. ADHD, traumatic brain injury) revealed discrepancies between WISC-III and WISC-IV scores and profiles. For example, children with attention deficit hyperactivity disorder (ADHD) showed greater index discrepancies on the WISC-IV, indicating that it might be better than the WISC-III in distinguishing strengths and weaknesses among this clinical population[4]. Thus, changes brought to the 4^th^ edition of the WISC might lead to a different pattern of subtest and index scores performance for autistic children as well.

With this mixed picture, the inclusion of intellectual functioning in the diagnosis leads to the crucial question on how to assess intelligence in autism, especially as some subtests and indexes seem more sensitive to certain neurodevelopmental conditions[39]. Thus, the composition of the instrument and the selection of subtests may have a greater impact for a clinical population than for typically developing individuals. Considering this variability, how will autistic individuals respond to different editions of the same instrument, namely two editions of the Wechsler scales? Does the cognitive profile remain stable despite changes within the test?

To date, only two studies have administered the WISC-IV to describe cognitive profiles on the autism spectrum. In a group of autistic children, the highest scores were on the two new reasoning subtests, *Matrix Reasoning* and *Picture Concepts*, and the lowest were on *Coding*, an attention subtest[4]. In another study, Oliveras-Rentas et al.[40] reported in a mixed group of ASD children strengths on the *Matrix Reasoning* and *Similarities* subtests, as well as weaknesses on the *Comprehension* subtest and two subtests comprising the Processing Speed Index (PSI), namely *Coding* and *Symbol Search*. In these studies, no direct comparison was made between the 3^rd^ and 4^th^ editions of the WISC.

These issues were addressed in the current study with three major goals. The first was to examine the specific WISC-IV index and subtest profile for a homogenous subgroup of ASD children, i.e. corresponding to DSM-IV autistic children. Given the changes made to the 4^th^ edition of the WISC, it was hypothesized that, in addition to the peak on the *Block Design* subtest, there would be a peak on the *Matrix Reasoning* subtest for this specific subgroup. We also compared the WISC-IV cognitive profile of two subgroups of autistic children, i.e. with and without speech onset delay. The second goal was to compare WISC-III and WISC-IV cognitive profiles, in order to verify whether the WISC-IV yields a more distinctive cognitive profile in autistic children. Based on previous research, it was hypothesized that the WISC-IV cognitive profile would differ significantly from the reported WISC-III autistic profile. Given the significant changes in the WISC-IV Perceptual Reasoning Index (PRI), it was expected that the discrepancy between the PRI and the Verbal Comprehension Index (VCI) would be more pronounced than on the previous edition, given that autistic children should get higher results on the WISC-IV PRI than on its previous counterpart, the PIQ. Finally, our third goal was to examine the impact of the WISC-IV on the cognitive profile of another subgroup, children with Asperger’s Syndrome, through an exploratory study. Previous studies indicate that their profile tends to differ from that of children with autism, specifically with higher results on the Verbal IQ compared to the Performance IQ of the WISC-III[21, 27, 41–46].

## Methods

### Ethics statement

Informed assent (child participants) and written informed consent (parents of child participants) was provided for any data included in the database, which was formally approved by the ethics committee of Rivière-des-Prairies Hospital (Montréal, Canada). The current project was also formally approved by the ethic committee of Rivière-des-Prairies Hospital (Montreal, Canada) (Project 13-14P).

### Participants

A total of 102 autistic and 84 typically developing children took part in this study. Firstly, all cases with WISC-IV FSIQ of 70 and higher were retrieved from the research database of the Specialized Autism Clinic at Rivière-des-Prairies Hospital (Montreal, Canada). From this initial selection, we retained all participants meeting the inclusion criteria described below for each group. This resulted in a sample of 51 autistic and 42 typical children, aged 6–16 years, who completed the WISC-IV. They were then individually matched on age and FSIQ scores to children having completed the WISC-III (see Tables 1 and 2). Matching groups based on FSIQ allowed comparing the distribution of subtest and index scores of both WISC editions.

**Table 1:** WISC-III cognitive profile.

	Autistic children		Asperger children		Typical children	
Sample size (sex)	51 (47M, 4F)		15 (12M, 3F)		42 (29M, 13F)	
	Mean	(SD)	Mean	(SD)	Mean	(SD)
Age	10.5	(2.6)	11.5	(3.2)	10.4	(2.4)
*Range*	*6–16*		*7–15*		*6–15*	
**WISC-III FSIQ**	90.6	(12.6)	99.4	(9.1)	102.8	(9.1)
***VIQ***	88.9	(15.2)	104.2	(8.0)	104.4	(9.2)
Similarities	9.6	(3.2)*	11.9	(2.2)**	11.4	(2.2)**
Comprehension	5.2	(3.0)*	7.6	(2.1)*	10.8	(2.6)
Vocabulary	7.2	(3.2)*	13.3	(3.2)**	10.9	(2.0)
Arithmetic	9.6	(4.1)**	11.1	(1.7)**	10.4	(2.5)
Digit Span	8.0	(3.6)	12.7	(2.0)**	8.9	(2.5)
***PIQ***	94.9	(12.0)	95.0	(14.2)	100.9	(11.4)
Block Design	12.2	(3.5)**	10.7	(3.0)	11.2	(2.3)**
Picture Completion	9.3	(2.9)	10.3	(3.2)	9.7	(2.0)*
Coding	6.8	(3.4)*	6.4	(3.0)*	8.9	(2.6)*
Object Assembly	9.0	(2.6)	8.5	(2.5)*	10.0	(2.7)
Picture Arrangement	8.2	(4.0)	9.9	(3.1)	9.5	(2.8)

WISC-III: Demographic characteristics and cognitive profile of autistic, Asperger and typical children who completed the WISC-III.

*Relative weakness.

**Relative Strength.

FSIQ: Full Scale Intelligence Quotient, VIQ: Verbal Intelligence Quotient, PIQ : Performance Intelligence Quotient

**Table 2:** WISC-IV cognitive profile.

	Autistic children		Asperger children		Typical children	
Sample size (sex)	51 (49M, 2F)		15 (12M, 3F)		42 (29M, 13F)	
	Mean	(SD)	Mean	(SD)	Mean	(SD)
Age	10.6	(2.7)	10.6	(2.6)	9.6	(2.3)
*Range*	*7–15*		*7–15*		*6–15*	
**WISC-IV FSIQ**	90.7	(12.4)	98.3	(12.4)	103.3	(13.5)
***VCI***	85.6	(16.1)	110.5	(10.5)	103.3	(16.3)
Similarities	8.4	(2.4)	12.5	(2.0)**	10.6	(3.3)
Comprehension	5.8	(3.0)*	9.3	(2.0)	9.2	(3.0)*
Vocabulary	8.2	(3.6)	13.3	(3.2)**	11.7	(3.5)**
***PRI***	105.8	(13.3)	101.3	(15.9)	105.2	(11.7)
Block Design	11.0	(3.1)**	9.5	(2.5)	9.9	(3.2)*
Matrix Reasoning	11.7	(2.9)**	10.4	(2.7)	10.8	(2.5)
Picture Concept	10.0	(2.8)**	10.5	(2.0)	11.6	(2.3)**
***WMI***	87.8	(16.9)	92.7	(15.0)	99.4	(12.6)
Digit Span	7.6	(3.3)*	8.3	(3.1)	9.2	(2.5)*
Letter-Number Sequencing	7.8	(3.2)*	9.3	(1.5)	9.2	(2.4)
***PSI***	91.5	(14.1)	85.2	(9.3)	101.6	(13.2)
Coding	7.7	(2.7)*	6.7	(1.9)*	10.2	(2.5)
Symbol Search	9.3	(3.3)	8.1	(2.0)*	10.5	(3.0)

WISC-IV: Demographic characteristics and cognitive profile of autistic, Asperger and typical children who completed the WISC-IV.

*Relative weakness.

**Relative Strength.

FSIQ: Full Scale Intelligence Quotient, VCI: Verbal Comprehension Index, PRI: Perceptual Reasoning Index, WMI: working Memory Index, PSI: Processing Speed Index.

#### Autistic children

Previous research found different patterns of cognitive abilities in autism spectrum individuals subgrouped according to speech development anomalies (i.e., in autistic versus Asperger subgroups) [47, 48]. For this reason, and for enhanced comparability within the groups and with previous studies, we chose to limit this study to autistic children as defined by the DSM-IV, i.e. ASD children characterized by speech delays **and/or** other speech development anomalies.

Thus, we retrieved data from all children who met criteria for the specific diagnosis of autism and who had completed the WISC-IV. From this sample, autistic children who had a known, diagnosable genetic syndrome or an additional neurological condition were excluded, leaving a non-syndromic or idiopathic autism group. It comprised ASD children with a speech delay (based on first single words after 24 months and/or first phrases after 36 months) and/or other language atypicalities during their development (echolalia, stereotyped language or pronominal reversal) as assessed in the Autism Diagnostic Interview-Revised (ADI-R; Lord, Rutter, Le Couteur, 1994). For most of the children, the diagnostic process combined expert interdisciplinary clinical judgment with two gold standard research diagnostic instruments, the ADI-R and the Autism Diagnostic Observation Scale-General (ADOS-G; Lord, Rutter, DiLavore & Risi, 1999). Among the102 autistic children, 86 were characterized by both ADI-R and ADOS-G, three by ADI-R only, nine by ADOS-G only, and four by direct observation based on the ADOS-G procedure and clinical interview based on ADI-R. All autistic participants who had ADI-R or ADOS-G scored above the cut-off for autism.

#### Exploratory study–Asperger children

An exploratory study was conducted with 15 children with an Asperger Syndrome (AS) diagnosis who completed the WISC-IV and 15 individually matched AS children who completed the WISC-III. Participants from the AS group presented no speech delay (first words at or before 24 months AND first phrases at or before 36 months). Among the 30 Asperger children, 26 were characterized by both ADI-R and ADOS-G, two by ADI-R only and two by ADOS-G only. Of the 28 Asperger participants who had ADI-R, 25 scored above the cut-off for autism and three scored slightly under (1 in ADI-Communication area and 2 in ADI-Social area). All Asperger participants who had ADOS-G scored above the cut-off for autism.

#### Typically developing children

Typically developing participants selected from the database were all recruited from the community (e.g. surrounding schools and community services) and had a typical academic background. Furthermore, they were screened using a semi-structured interview; participants with a personal or family (1^st^ degree) history of psychiatric, neurological or other medical conditions potentially affecting brain development were identified and their data were excluded.

### Measures

The WISC is an individually-administered test battery that assesses intelligence in school-age children (6 years to 16 years 11 months). **The Wechsler Intelligence Scale for Children– 3** ^**rd**^ **edition**[17]comprises 10 core subtests that compute VIQ and PIQ, which, when combined, yield the FSIQ (*M* 100 and *SD* 15). The Verbal scale includes *Vocabulary*, *Similarities*, *Comprehension*, *Arithmetic* and *Digit Span* subtests, whereas the Performance scale includes *Block Design*, *Picture Arrangement*, *Picture Completion*, *Object Assembly* and *Coding* subtests (for all subtests *M =* 10 and *SD =* 3).

The **4** ^**th**^ **edition of the Wechsler Intelligence Scale for Children**[37] comprises 10 core subtests, yielding four index scores that combine into the FSIQ. The Verbal Comprehension Index (VCI) is similar to the previous VIQ and consists of three of the previous five 3^rd^ edition subtests (*Similarities*, *Comprehension* and *Vocabulary*). The PRI presents more changes when compared to the previous PIQ. Specifically, the PRI still comprises *Block Design* but adds two new subtests, *Matrix Reasoning* and *Picture Concepts*. Only one of these tasks is timed and implies the use of materials (*Block Design*) compared to four subtests of the WISC-III PIQ. The Working Memory Index (WMI; *Digit Span* and *Letter-Number Sequencing*) and the Processing Speed Index (PSI; *Coding* and *Symbol Search*) now represent distinct indexes, as well as being part of the FSIQ. Index standard scores have a mean of 100 and a standard deviation of 15.

The WISC was administered by a clinical psychologist using Canadian norms, and the final diagnosis was not available at the moment of testing.

### Data analysis

Within each group, repeated-measures analysis of variance (ANOVA) assessed discrepancies across indexes. Relative “strengths” and “weaknesses” at the group level were analyzed by pair-wise *t* tests comparing scores on a specific subtest with average performance on all 10 subtests. “Peaks” and “troughs” were computed on an individual basis when the difference between a participant’s score on a subtest minus his/her mean subtest performance exceeded the threshold specified in Wechsler’s manual (corresponding to a difference seen in less than 5% of the normative sample). Performance on corresponding indexes in both WISC editions was contrasted by repeated-measures ANOVA within each group.

Data were analyzed with SPSS 21.0, with a significance level of *p*≤.05 (corrected for multiple comparisons with a Bonferroni correction, where applicable). Effect sizes are reported as eta squared for analyses of variance and Cohen’s *d* effect size for pair-wise t tests.

## Results

The group of autistic children having completed the WISC-III did not differ from the one having completed the WISC-IV on age (*p* = .912) and FSIQ (*p* = .975). Both groups ranged from 6 to 16 years of age (*M* = 10 years for both groups). Nearly half (55%) of the autistic sample presented a speech onset delay, while the second half did not but showed language atypicalities during early development.

No significant difference of age (*p* = .226) or FSIQ (*p* = .956) was found between TD children having completed the WISC-III and the WISC-IV. Both groups ranged from 6 to 15 years of age with a mean age of 10 years old.

Finally, no significant age difference was found when comparing autistic and TD children (*p* = .386). There was however a significant difference between autistic and typical children on WISC-IV FSIQ (*p*< .001).

### WISC-IV cognitive profile

#### Autistic group

**Fig 1:**
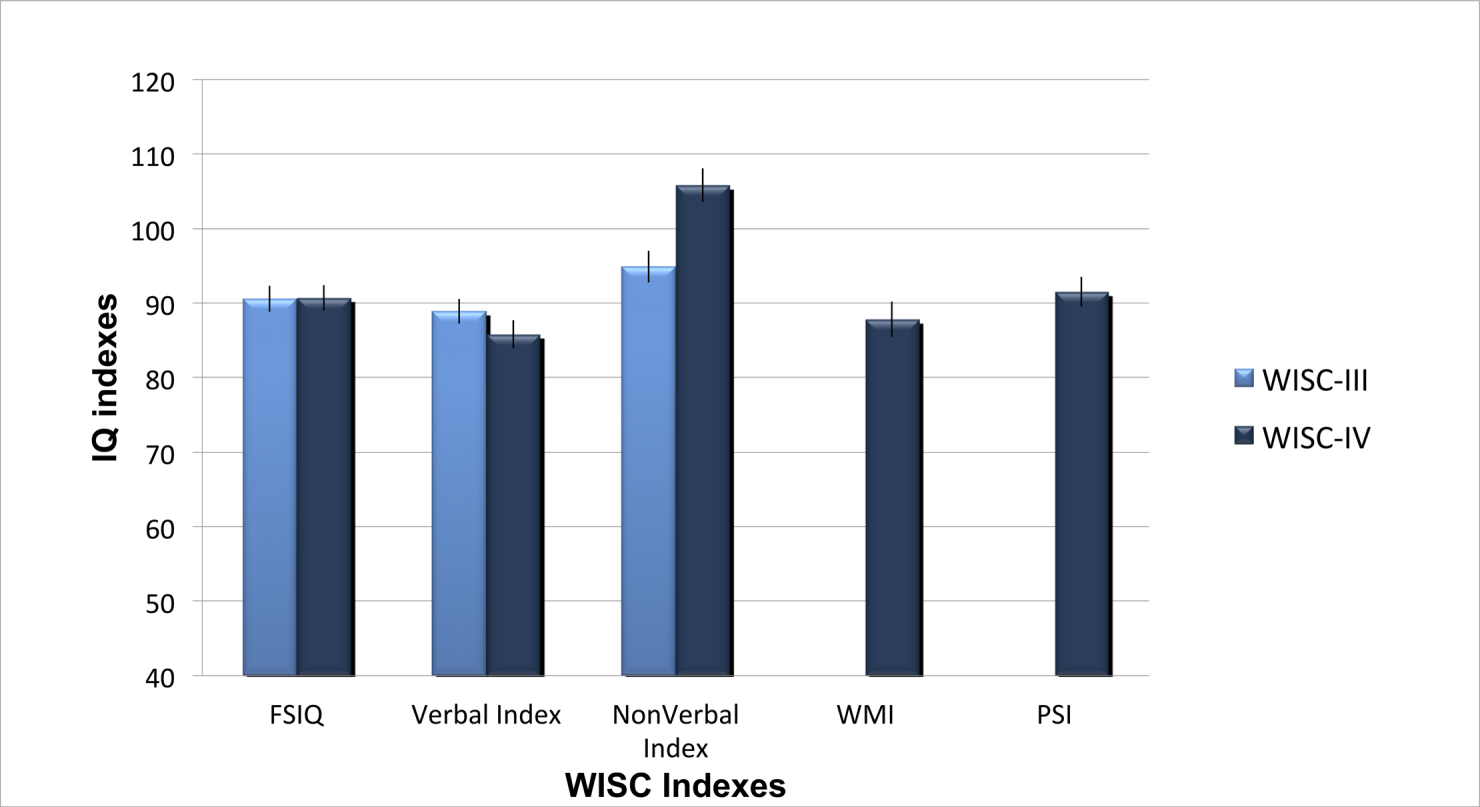
Autistic WISC-III and -IV IQ indexes. Mean standard scores on WISC-III and WISC-IV indexes. FSIQ: Full Scale IQ; Verbal: Verbal Comprehension Index in WISC-IV and Verbal Index in WISC-III); NonVerbal IQ: Perceptual Reasoning Index in WISC-IV and Performance IQ in WISC-III; WMI: WISC-IV Working Memory Index; PSI: WISC-IV Processing Speed Index. Error bars represent standard error from the mean.

The scores of autistic children on the FSIQ and on each of the four indexes differed significantly, *F* (4, 200) = 23.98, *p* < .001, *n* ^2^ = .32. The PRI was significantly higher than the FSIQ and than the other three indexes (all *p* < .001). There was no difference between the VCI, the WMI and the PSI (all p>0.05), although the VCI was significantly lower than the FSIQ (*p* = .021) (see Table 2 and Fig 1).

The majority (48/51 or 94%) of autistic children had a higher PRI than VCI score, and 35/51 (69% of the sample) had at least a 12-point difference in favor of the PRI (12 points being considered a statistically and clinically significant difference between IQ subscales; Sattler, 1992) (see Table 3).

**Table 3:** Discrepancies between WISC-IV indexes.

	Autistic children	Asperger children	Typical children
**PRI > VCI**	48/51 (94.1%)	4/15 (26.7%)	25/42 (59.5%)
*Greater than 12 points*	35/51 (68.6%)	1/15 (6.7%)	9/42 (21.4%)
**VCI > PRI**	3/51 (5.9%)	11/15 (73.3%)	17/42 (40.5%)
*Greater than 12 points*	1/51 (2.0%)	7/15 (46.7%)	9/42 (21.4%)
**VCI > PSI**	19/51 (37.3%)	14/15 (93.3%)	24/42 (57.1%)
*Greater than 12 points*	9/51 (17.6%)	12/15 (80.0%)	10/42 (23.8%)

Proportion of discrepancies between Verbal Comprehension Index (VCI), Perceptual Reasoning Index (PRI) and Processing Speed Index (PSI) among autistic, Asperger and Typically Developing children.

Some specific strengths and weaknesses were revealed in the autistic group when comparing performance on the different subtests. Significant strengths were observed on the *Block Design* (*t* = 5.5, *p* < .001, *d* = .95),*Matrix Reasoning* (*t* = 9.2, *p* < .001, *d* = 1.29) and *Picture Concept*s (*t* = 4.0, *p* < .001, *d* = .57)subtests when compared to the average performance on all subtests. Indeed, at the individual level, 33% of autistic children showed a peak in their performance on the *Block Design* subtest (e.g. difference between *Block Design* score and mean score on all subtests greater than that found in less than 5% of the Wechsler normative sample), while 49% presented a peak in their performance on the *Matrix Reasoning* subtest. At the group level, significant weaknesses were noted on the following subtests: *Comprehension* (*t* = 9.32, *p* < .001, *d* = 1.27), *Digit Span* (*t* = 2.93, *p* = .005, *d* = .49), *Letter-Number Sequencing* (*t* = 2.47, *p* = .02, *d* = .38) and *Coding* (*t* = 2.67, *p* = .01, *d* = .46).Individually, 35% of autistic children exhibited a trough on the *Comprehension* subtest (e.g. difference between the *Comprehension* score and mean score on all subtests greater than that found in less than 5% of the Wechsler normative sample),while 27% displayed a trough on the *Coding* subtest.

**Fig 2:**
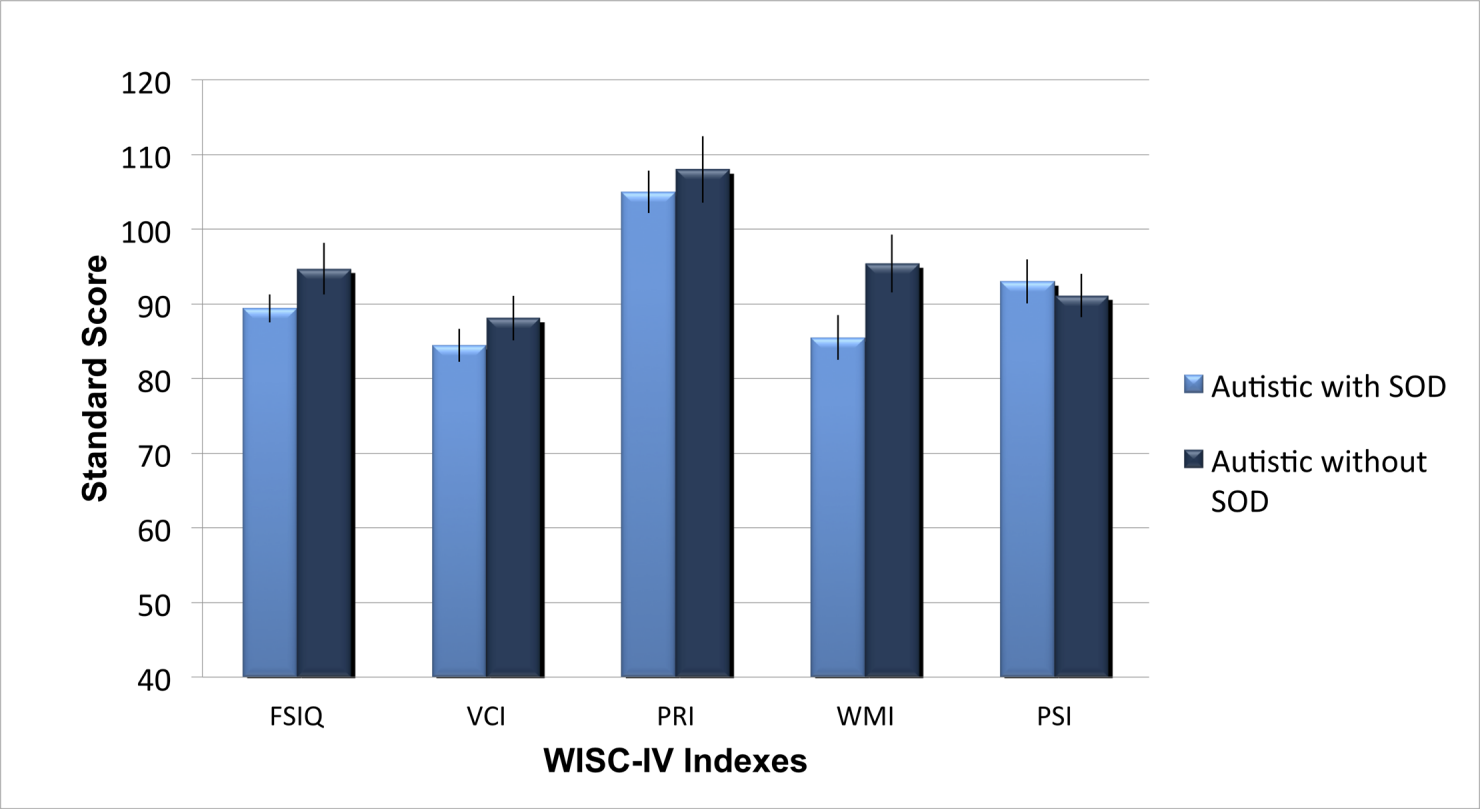
WISC-IV profile of autistic children with and without speech onset delay. Mean standard score on WISC-IV comparing autistic with and without speech onset delay (SOD). FSIQ: Full Scale IQ, VCI: Verbal Comprehension Index, Perceptual Reasoning Index, WMI: Working Memory Index, PSI: Processing Speed Index. Error bars represent standard error from the mean.

In a further analysis, we explored the impact of speech onset delay in the autistic group. More than half of the autistic sample presented a speech onset delay (n = 28/51) while a third did not (n = 18/51) (5 children with unavailable information). Complementary analysis revealed no significant difference on the cognitive profile for those two subgroups, all *p*>.05 for the FSIQ, the PRI, the VCI and the PSI. Only the WMI differed between both groups and appears lower for the autistic children with a speech onset delay (95.44 vs. 85.50, *p* = .02) (see Fig 2).

We also further explored the role of age in our autistic group. Splitting the WISC-IV autistic sample based on age yielded two equal subgroups: 25 children ranging from6-10 years of age and 26 children ranging from11-15 years of age. Comparing both subgroups on their cognitive profiles revealed similar results on their FSIQ, VCI, WMI and PSI (all p >0.05) but a significant difference on their PRI (*p* = .02). Indeed, younger children tended to score higher on the PRI (*M* = 110.24, *SD* = 13.78) than older children (*M* = 101.54, *SD* = 14.51). However, the advantage of the PRI over the VCI reached a significant level for both subgroups (both *p* < .001).

#### Exploratory study—Asperger group

**Fig 3:**
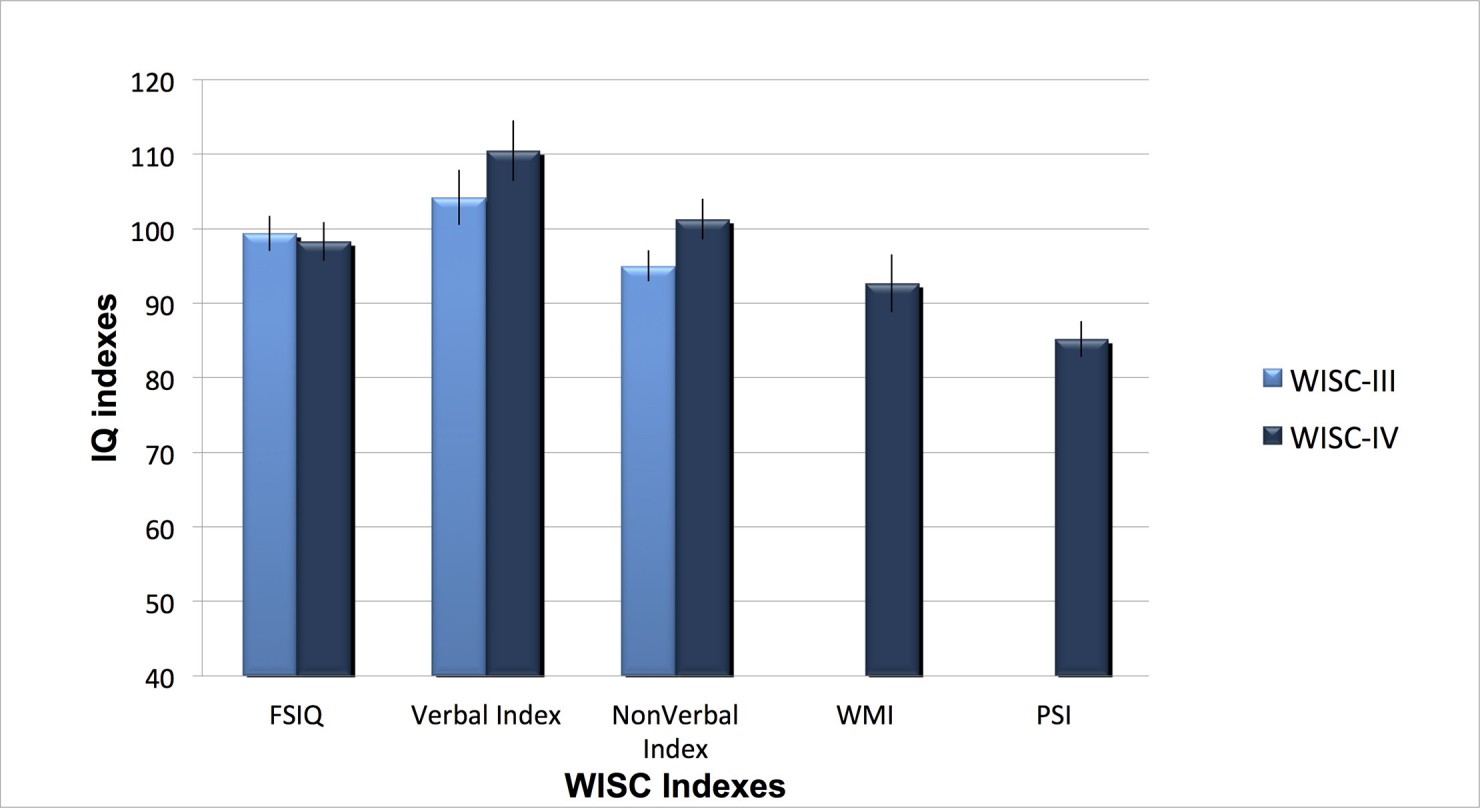
Asperger WISC-III and -IV IQ indexes. Mean standard scores on WISC-III and WISC-IV indexes. FSIQ: Full Scale IQ; Verbal: Verbal Comprehension Index in WISC-IV and Verbal Index in WISC-III); NonVerbal IQ: Perceptual Reasoning Index in WISC-IV and Performance IQ in WISC-III; WMI: WISC-IV Working Memory Index; PSI : WISC-IV Processing Speed Index. Error bars represent standard error from the mean.

The FSIQ and the performance on each of the four indexes differed significantly in the Asperger group, *F* (4, 56) = 12.51, *p* < .001, *n* ^2^ = .472. Their best performance was on the VCI, on which they scored higher than on the PSI (*p*<0.01), the WMI (*p* < .001) and the PRI (*p* = .06), though this latter result did not reach significance. The PRI was also significantly higher than the PSI (*p* < .05) (see Table 2 and Fig 3).The majority of Asperger children (11/15 or 73%) scored higher on the VCI than on the PRI, and seven (47%) showed a difference of at least 12 points between the two index scores (see Table 3).

In contrast to the autistic group, the strengths of Asperger children were among the verbal subtests. At the group level, significant strengths were noted on the *Vocabulary* (*t* = 5.1, *p*<0.01, *d* = 1.57) and *Similarities* (*t* = 5.4, *p*<0.001, *d* = 1.66) subtests. At the individual level, 50% of Asperger children showed a significant peak of performance on the *Vocabulary* subtest and 29% on *Similarities* subtest. Significant weaknesses were seen on the *Digit Span* (*t* = 2.70, *p* < .05, *d* = .72) and *Coding* (*t* = 5.3, *p* < .001, *d* = 1.97) subtests. Indeed, 53% of Asperger children displayed a trough on the *Coding* subtest.

#### Typically developing group

As opposed to the other two groups, there were no significant discrepancies between the four main indexes for typical children, *F* (4, 164) = 2.35, *p*>.05, *n* ^2^ = 0.06 (see Tables 2 and 3). As expected, significant performance peaks were much less frequent in the typically developing group than in the other groups. Although the Picture Concept and Vocabulary subtests represented significant strengths at the group level, they were less frequent than seen in the autistic group (Picture Concept: *p* = .026, 10% of the sample; Vocabulary: *p*<0.01, 23.8% of the sample). Again, even though Comprehension displays a trough at the group level, *p* = 0.006, this trough was present in only 8% of the sample.

### Comparison between WISC-III and WISC-IV profiles

#### Autistic group

Autistic children’s WISC-III profile displayed discrepancies between the FSIQ, the VIQ and the PIQ, *F* (2, 100) = 7.33, *p* = .001, *n* ^2^ = .128. Their PIQ was significantly higher than their VIQ (p = .01) and their FSIQ (p = .002). Specific strengths and weaknesses were revealed among autistic children who completed the 3^rd^ edition. Specifically, they exhibited strengths on the *Block Design* (*p* < .001) and *Arithmetic* (*p* = .03) subtests, and weaknesses on the *Vocabulary* (*p* < .001),Similarities (p = .02),*Comprehension* (*p* < .001)and *Coding* (*p* = 0.001) subtests.

Comparison of both editions disclosed a 11-point difference in favor of the WISC-IV PRI when compared to WISC-III PIQ (*t* = 4.35, *p* < .001, *d* = .86), for the same mean FSIQ. As results on the VIQ remained stable on both editions (*p* = .29), the discrepancy between verbal and nonverbal scores has more than tripled on the WISC-IV (PRI > VCI) when compared to the WISC-III (PIQ > VIQ; *t* = 4.29, *p <* .001, *d* = .42)(see Fig 1).

#### Exploratory study—Asperger group

As expected, we observed significantly higher WISC-III VIQ than PIQ in Asperger children (*t* = 2.29, *p* = .038, *d* = 0.83). Analysis of specific strengths and weaknesses also showed particular strengths on the *Information* (*p* < .001),*Similarities* (*p* = .001)and *Vocabulary* (*p* = .03) subtests. Performance on the *Coding* (*p* < .001), *Object Assembly* (*p* = .02) and *Comprehension* (*p* = .004) subtests appeared as significant weaknesses.

When comparing the WISC-III to the WISC-IV, we noted higher WISC-IV VCI scores than WISC-III VIQ score, though the difference was not statistically significant (*p* = .08). The discrepancy level between scores on the Verbal and Nonverbal scales was equivalent in both WISC editions (*p* = 0.9)(see Fig 3).

#### Typically developing group

Unlike the other two groups, WISC-III VIQ and PIQ scores in typically developing children were not significantly different, *t* = 1.4, *p* = .17. Some significant strengths (*Similarities*, *p =* .*001*, and *Block Design* subtests, *p* = .01) and weaknesses (*Coding* subtest, *p* = .05 and Picture Completion, *p* = .04) were noted at the group level, but were smaller than in the other two groups. As expected, comparing PRI and PIQ scores, as well as VCI and VIQ scores, did not reveal any significant differences for typically developing children.

## Discussion

### Results summary

This study compared the cognitive profiles of children with an autism diagnosis as described by the DSM-IV (i.e. children with speech onset delay and or atypicalities in their language development) using the WISC-III and the WISC-IV. Overall, the WISC-IV profile was consistent with the one obtained with the WISC-III, though discrepancies between subscale scores were more pronounced on the latest edition. Autistic children scored higher on the PRI than on the other three WISC-IV indexes. Additionally, the gap between the verbal and the non verbal subtests has more than doubled on the WISC-IV. Lastly, a peak on the new *Matrix Reasoning* subtest emerged. In contrast to the clinical group, the scores of typically developing children did not differ significantly on all four indexes.

An exploratory study with a different subgroup of the ASD continuum, i.e. children with Asperger’s Syndrome as defined by the DSM-IV, yielded a similar cognitive profile with both the WISC-III and the WISC-IV. The Asperger children’s performance was not affected by the changes made to the PRI the same way as was the autistic children’s performance. The gap between verbal and non verbal subtests remained the same between both WISC editions in Asperger children.

### WISC-IV autistic cognitive profile and differences from WISC-III

Although the present results are consistent with previous findings, interestingly, the PRI advantage over the VCI in autistic children has more than doubled on the WISC-IV compared to the PIQ over VIQ advantage on the WISC-III. The PRI’s latest configuration resulted in significantly higher scores for autistic children. Two new visual reasoning tasks, *Matrix Reasoning* and *Picture Concepts*, were added and some timed visual-motor subtests were withdrawn. Thus, in autistic children, visual-spatial and reasoning strengths were more apparent on the WISC-IV, likely in part because the nonverbal subtests of WISC-III relied more heavily on motor coordination and speed.

Consistent with their higher PRI scores than on any other index, nearly half of the autistic children presented a clinically significant performance peak on the *Matrix Reasoning* subtest, and a third of them on *Block Design* subtest. These results are similar to those reported in previous WISC-IV studies, in which autistic children’s performance peaked on the *Matrix Reasoning* subtest[4, 40]. In accordance with previous WISC-III and WISC-IV studies, performance on the *Comprehension* subtest, which requires language comprehension abilities and social reasoning, remained the lowest for the autistic group[4, 28, 31].

Our results may contribute to further understand how intelligence takes place in autism. Indeed, peaks of abilities, once considered as isolated areas of strength, are increasingly considered as manifestations of genuine intelligence. Hence, autistic children demonstrate a strong performance on the *Matrix Reasoning* subtest, which is in accordance with previous findings stating that autistic children and adults would score significantly higher on *Raven’s Progressive Matrixes* than predicted by their WISC-III and WISC-IV FSIQs [14, 49, 50]. The WISC-IV’s *Matrix Reasoning* subtest and *Raven’s Progressive Matrices* both assess high-level reasoning using nonverbal geometric stimuli, and require the ability to infer rules while generating and maintaining goals in working memory. The same is true of autistic children’s strength on the *Picture Concept* subtest, which requires the ability to infer and reason using available semantic relationships between stimuli. The fact that all three PRI subtests represent relative strengths for autistic children challenges the idea that autistic strengths and intelligence are merely a collection of simple, low-level perceptual abilities. In the same way, Stevenson and Gernsbacher[51] found that while autistic and non-autistic participants performed equally well in a range of reasoning tasks, autistic participants outperformed non-autistic participants on abstract spatial reasoning tests.

In the past years, multiple studies have highlighted the visuospatial peaks of performance typically seen in autistic children. Behavioural evidence such as superior pattern matching, pattern construction, and mental rotation of visuospatial information[26, 51, 52] have repeatedly been reported among high-functioning autistic individuals. These behavioral findings are consistent with fMRI studies showing that autistic individuals, as compared to non-autistic individuals, displayed greater activity in visual cortex while solving a variety of visuospatial tasks[53, 54].

### Exploratory study—WISC-IV Asperger profile

As reported by previous studies, Asperger children’s cognitive profile indicates relative strengths on verbal subtests. Indeed, one out of two Asperger participants presented a clinically significant performance peak on the *Vocabulary* subtest; and one out of three presented a peak performance on the *Similarities* subtest. Earlier studies have revealed higher scores for Asperger than for autistic individuals on various language measures[42, 55, 56]. Previous results were however confounded by higher FSIQ scores in the Asperger group when compared to autistic children.

Another distinctive characteristic of Asperger children’s WISC-IV profile is their relatively low PSI score and its significant discrepancy with VCI score. Their lowest scores were on the *Coding* and *Symbol Search* subtests, two timed tasks that require visual-motor coordination. In fact, their PSI was significantly lower than that of typically developing children, despite their similar FSIQ scores. Lower PSI scores than on any other index were also noted in earlier WISC-IV studies of autism spectrum children[4, 40]. Can this be attributed to slower processing speed in Asperger children, as was previously suggested? Contrary to this interpretation, Asperger individuals have recently been found to have processing speed abilities in accordance to their Wechsler IQ, as measured by an Inspection Time task [57, 58]. Inspection Time task assesses processing speed without relying on timed motor responses, contrary to the requirement of the Wechsler PSI. Indeed, some studies have found motor impairment in Asperger individuals[59, 60], as well as greater difficulties with manual dexterity than in autistic children[61]. The well-documented motor coordination weaknesses[62, 63] combined with attention difficulties[60, 64, 65] are likely to account for Asperger children’s low performance on the PSI.

Asperger children did not seem to benefit as much as autistic children from the changes introduced in the new PRI, as their results on this index were very similar to those obtained with the WISC-III PIQ. These results are in accordance with the less significant discrepancy found in Asperger children when comparing their scores on *Raven’s Progressive Matrixes* and WISC-III[47]. These differences could be explained by Asperger individuals’ greater reliance on verbal rather than visual-spatial processing. Indeed, autistic participants have previously been found to favor visual-spatial strategies over linguistic ones, when solving matrix reasoning problems, in contrast to Asperger and typically developing participants who favored the latter[66].

### Challenges related to the assessment of intelligence in autism

The present results are consistent with DSM-5, which states the importance of defining the cognitive profile of the individual with an autism spectrum diagnosis. DSM-5 proposes the use of different specifiers to describe individual profile, which include the specification of “with or without accompanying intellectual impairment” and “with or without accompanying language impairment”. Studying cognitive profiles in autism spectrum children highlights the heterogeneity of this population.

Firstly, as found in several previous studies, there was greater within-subject heterogeneity in autism spectrum children than in typically developing children. In the current study, significant discrepancies between at least two of the four indexes were encountered in a majority of our clinical sample (64%).This discrepancy seemed to vary according to the age of the child; the gap between the indexes tends to be more representative of younger autistic children and less prominent in adolescence. This result is consistent with previous findings of a NVIQ>VIQ profile occurring more frequently in younger than older male autistic children[67]. Additional studies are needed in order to explore the issue of age on cognitive profiles among the autism spectrum.

Heterogeneity is also observed across the autism spectrum as patterns of performance on WISC-IV indexes and subtests greatly vary between ASD children. A subgroup of ASD children, namely the one corresponding to autism as defined by the DSM-IV, seems to involve greater visual than verbal reasoning skills, while others, such as children characterized by the DSM-IV definition of Asperger’s Syndrome, present greater verbal strengths and lower processing speed scores. Nonetheless, this variability across indexes may be useful in identifying subtypes of ASD children that differ regarding variables such as language development atypicalities. Patterns of performance and discrepancies among subtests and indexes are likely to offer greater information for differential diagnoses and intervention tools. The WISC-IV cognitive profile could contribute to screening and diagnosing autism.

A second issue related to the question of assessing intelligence in autism concerns the structure and the content of the selected instrument. In the present study, the advantage of the PRI over the PIQ was not observed in typically developing children; both measures yielded equivalent scores. These results emphasize the idea that content changes differentiating two editions of the same tests are susceptible to have a greater effect on a clinical population than on a normative one. Furthermore, this gap between both editions could have major impact in a clinical settingwhen clinicians are asked to state the intellectual functioning of a child. Do lower results on Wechsler scales reflect the actual cognitive abilities of the child or rather a difficulty adjusting to the content of the assessment? As an example, the 11-pointdifference between the WISC-IIIPIQ and the WISC-IV PRI found in the autistic group, could make the difference between a child considered to have an intellectual disability on WISC-III and one having low average intelligence on WISC-IV. Therefore, autistic individuals might be more sensitive to the choice of subtests used to assess intelligence, and might be more likely to respond differently according to the type of task used in the test. With the arrival of a new edition of the WISC this year, it will be interesting to follow how autistic children respond to the test’s new configuration.

## Conclusion

With the actual DSM-5 and related changes in nomenclature, the present results act as a reminder that specific cognitive profiles exist among the autism spectrum and therefore, justify the need for neuroscientists to better understand these different subgroups. Using additional criteria (e.g. onset speech delay, cognitive strengths) to obtain less heterogeneous groups would help in pinpointing different trajectories characterizing the autism spectrum as well as the underlying genetic and neural mechanisms. The description and comprehension of cognitive strengths and weaknesses among subgroups on the autism spectrum also have implications for educational interventions. Indeed, they may contribute to the development of learning strategies building on the particular cognitive strengths of each subgroup.
